# Definitive Diagnosis of Abdominal Tuberculous Lymphadenitis by Endoscopic Ultrasound-Guided Tissue Acquisition and Bacterial Culture: A Case Report

**DOI:** 10.1159/crg/abwag003

**Published:** 2026-08-31

**Authors:** Maiko Morii, Akie Yoshikawa-Kimura, Akinobu Nakata, Yoshinori Shimamoto, Hirotsugu Maruyama, Yu Nishida, Masaki Ominami, Yuji Nadatani, Shusei Fukunaga, Koji Otani, Shuhei Hosomi, Fumio Tanaka, Masahiro Kato, Masanori Shiohara, Yasuhiro Fujiwara

**Affiliations:** a Department of Gastroenterology, Osaka Metropolitan University Graduate School of Medicine, Osaka, Japan; b Department of Pathology, Osaka Metropolitan University Graduate School of Medicine, Osaka, Japan

**Keywords:** Tuberculous lymphadenitis, Endoscopic ultrasound-guided tissue acquisition, Bacterial culture

## Abstract

**Introduction:**

Intra-abdominal lymphadenopathy presents a diagnostic challenge. Tuberculous lymphadenitis is an important differential diagnosis as several noninfectious conditions can mimic its presentation. Although endoscopic ultrasound-guided tissue acquisition (EUS-TA) is a minimally invasive diagnostic tool, combining multiple examinations – such as Ziehl-Neelsen staining, bacterial culture, and polymerase chain reaction – is essential for accurate diagnosis due to the paucibacillary nature of extrapulmonary tuberculosis. We report a case of abdominal tuberculous lymphadenitis diagnosed through EUS-TA and bacterial culture.

**Case Presentation:**

A 66-year-old Japanese woman had experienced a fever lasting several weeks and a weight loss of 2 kg over the past year. Computed tomography (CT) revealed multiple lymphadenopathies in the mediastinum and abdominal cavity. We performed EUS-TA on a hypoechoic lymphadenopathy in the peripancreatic region to differentiate among infectious, inflammatory, and neoplastic causes of lymphadenopathy. Histopathological examination revealed the presence of epithelioid cell granulomas, indicative of tuberculosis. An interferon-γ release assay was subsequently performed and found to be positive, prompting the second EUS-TA to obtain samples for the identification of *Mycobacterium tuberculosis*. The patient was diagnosed with abdominal tuberculous lymphadenitis based on the cumulative findings. The patient improved clinically after 4 months of antituberculous therapy, accompanied by a marked reduction in the lymphadenopathies on follow-up CT.

**Conclusion:**

This case highlights the importance of incorporating bacterial culture, in addition to histopathological examination, as an essential diagnostic tool for tuberculous lymphadenitis.

## Introduction

Intra-abdominal lymphadenopathy is a common diagnostic challenge faced by clinicians [1]. Since several infectious and noninfectious diseases can mimic the same clinical picture, tuberculous lymphadenitis should be included in the differential diagnosis. Endoscopic ultrasound-guided tissue acquisition (EUS-TA) has proven to be a valuable, minimally invasive alternative to surgical procedures for the diagnosis of abdominal tuberculosis [2]. However, since the extrapulmonary forms of tuberculosis are paucibacillary, the diagnostic yield of these techniques may be low and is based on the quality of the sample obtained. A combination of diagnostic modalities, including Ziehl-Neelsen staining, mycobacterial culture, and polymerase chain reaction (PCR), is essential for arriving at an accurate diagnosis. In this report, we describe a case of abdominal tuberculous lymphadenitis diagnosed by EUS-TA and bacterial culture, highlighting the importance of bacterial culture in cases with inconclusive findings from other diagnostic modalities. The CARE Checklist has been completed by the authors for this case report, attached as online supplementary material (for all online suppl. material, see https://doi.org/10.1159/crg/abwag003).

## Case Report

A 66-year-old Japanese woman presented to our hospital with a fever lasting several weeks and a weight loss of 2 kg over the past year. Computed tomography (CT) revealed multiple lymphadenopathies in the mediastinum and abdominal cavity. The patient was referred to our hospital for further evaluation and treatment. She had been receiving ursodeoxycholic acid for primary biliary cholangitis at our hospital.

On admission, her vital signs were within normal limits, and the physical examination revealed no other abnormal findings. Laboratory test results were largely within normal limits, including tumor markers such as carcinoembryonic antigen, carbohydrate antigen 19-9 (CA19-9), and s-pancreas antigen-1 (SPan-1). However, the soluble interleukin-2 receptor (sIL-2R) level was mildly elevated at 706 U/mL.

Contrast-enhanced CT revealed lymphadenopathy in the mediastinum, para-aortic region, and around the pancreas, with central areas showing poor enhancement (Fig. 1a). Fluorodeoxyglucose positron emission tomography-CT scan revealed increased fluorodeoxyglucose uptake in the lymph nodes in the peritracheal region, right pulmonary hilum, para-aortic region, lesser curvature of the stomach, and pancreas (Fig. 1b). Esophagogastroduodenoscopy and colonoscopy revealed no evidence of malignancy.

**Fig. 1.**
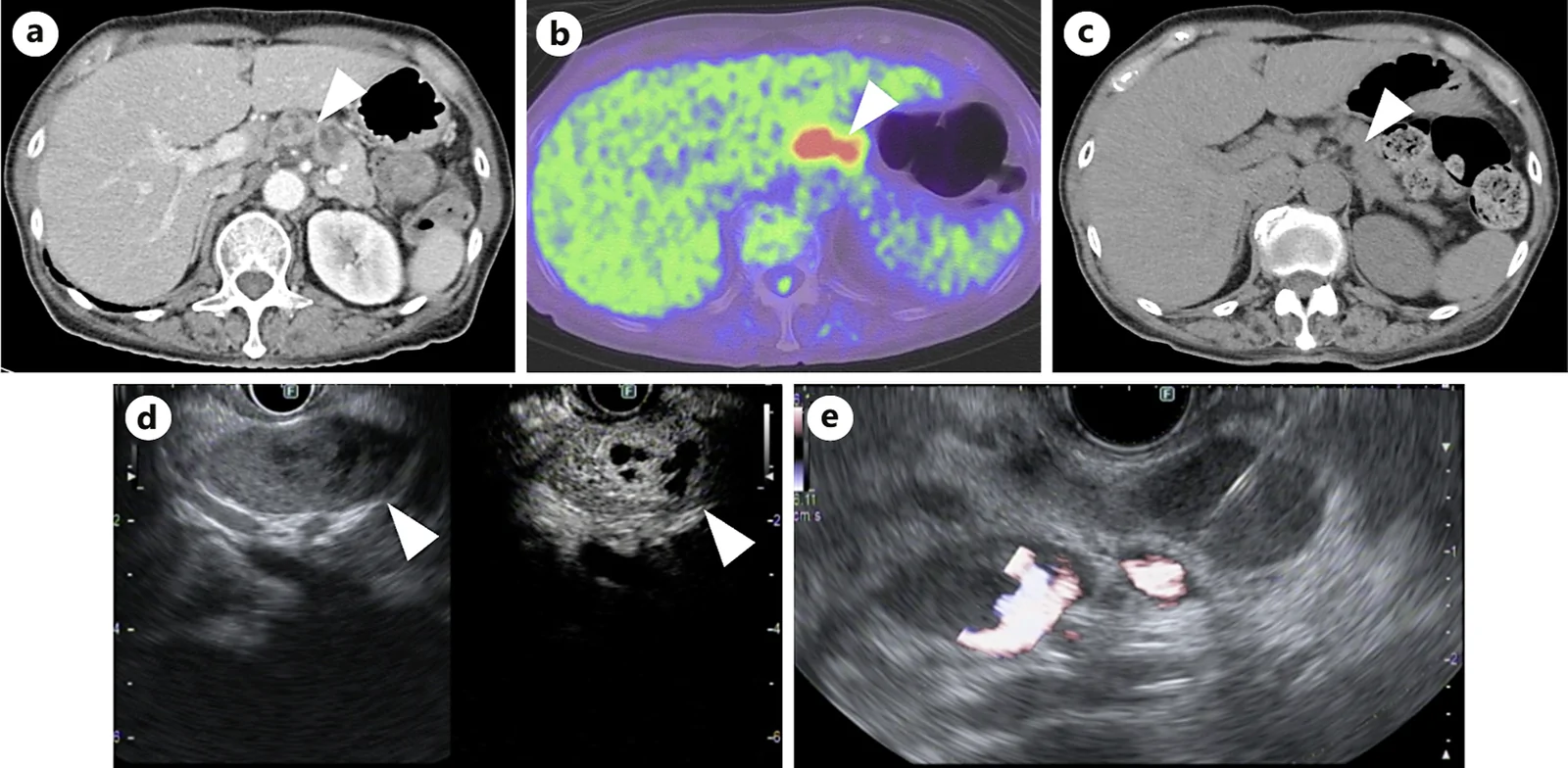
**a** Contrast-enhanced CT shows multiple low-density masses around the pancreas (arrow). **b** Fluorodeoxyglucose positron emission tomography (FDG-PET) shows increased FDG uptake in the pancreatic body (arrow). **c** Follow-up CT shows marked reduction in the size of the lymphadenopathies (arrow). **d** Endoscopic ultrasonography (EUS) shows multiple hypoechoic and heterogeneous lymphadenopathies in the peripancreatic region, and contrast-enhanced EUS shows early enhancement except in the central area (arrow). **e** EUS-TA was performed on a 37 × 19 mm hypoechoic lymphadenopathy.

Endoscopic ultrasonography (EUS) demonstrated multiple well-demarcated, hypoechoic, and heterogeneous lymphadenopathies with a mosaic-like internal echo pattern in the peripancreatic region and around the tracheal carina. Contrast-enhanced EUS with perflubutane revealed early enhancement, sparing the central area (Fig. 1d). Based on these findings, we considered a broad differential diagnosis, including infectious, inflammatory, and neoplastic etiologies of lymphadenopathy.

EUS-TA on the lymphadenopathies around the tracheal carina was considered unfeasible due to the inability to avoid the necrotic areas. Therefore, EUS-TA was performed on a 37 × 19 mm hypoechoic lymphadenopathy in the peripancreatic region using a 22-gauge SharkCore needle (Fig. 1e). Histopathological examination revealed epithelioid cell granulomas and necrosis without evidence of malignancy (Fig. 2a, b). Ziehl-Neelsen staining was negative for *Mycobacterium* spp. (Fig. 2c). The presence of necrotizing granulomas raised the possibility of granulomatous disease, including tuberculous lymphadenitis and sarcoidosis. Therefore, the QuantiFERON-TB Gold In-Tube (QFT) test was performed, which was subsequently found to be positive. Based on this result, we performed EUS-TA again on the same lymphadenopathy using the same 22-gauge SharkCore needle to obtain samples for PCR and bacterial culture.

**Fig. 2.**
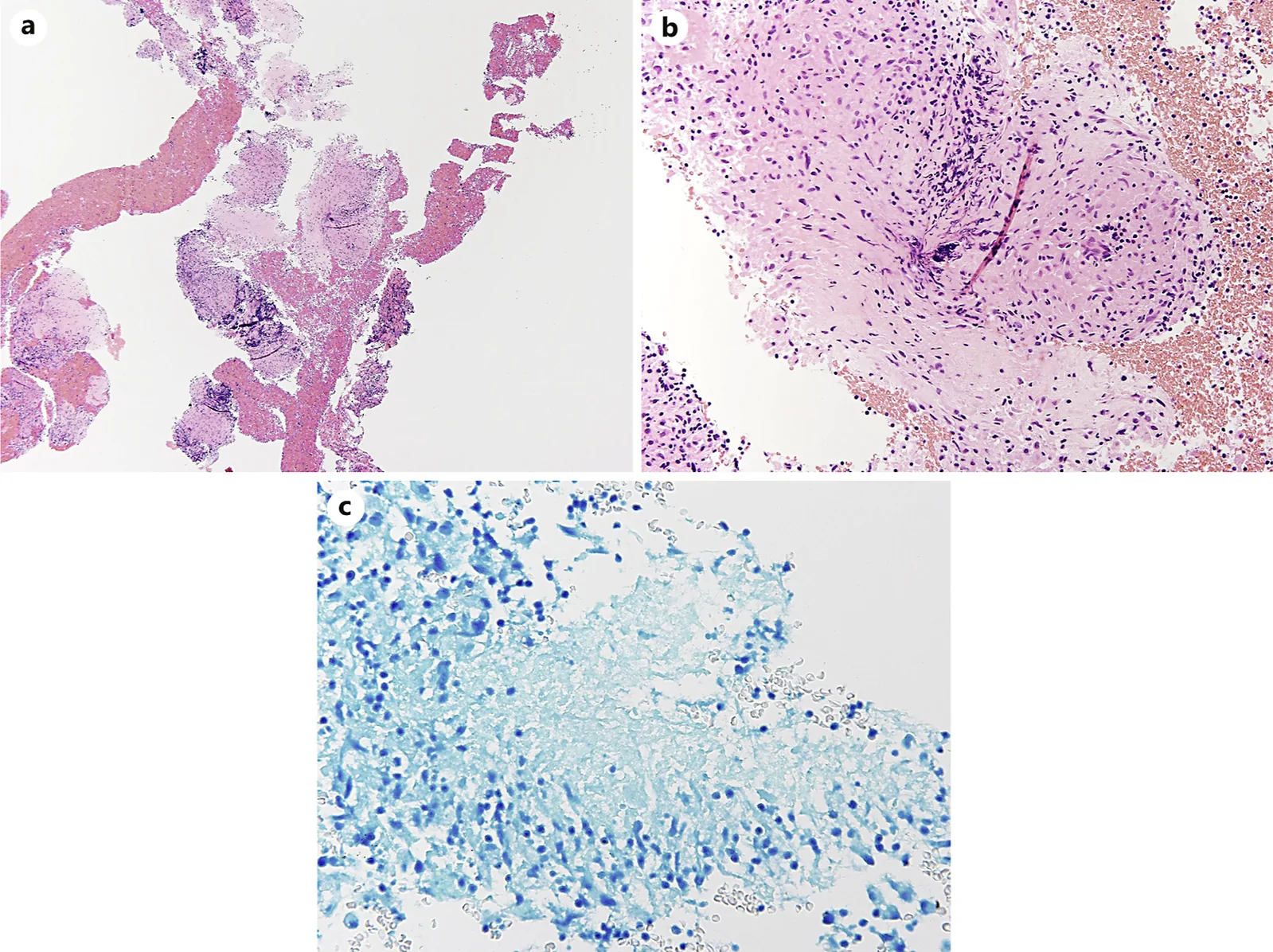
Histopathological findings of EUS-TA samples showing epithelioid cell granulomas and necrosis (**a**: hematoxylin and eosin stain, ×40; **b**: hematoxylin and eosin stain, ×400), with no acid-fast bacilli detected (**c**: Ziehl-Neelsen stain).

Ziehl-Neelsen staining and PCR did not demonstrate the presence of tuberculosis; however, bacterial culture was positive for *M. tuberculosis*. The patient was diagnosed with tuberculous lymphadenitis, and treatment with isoniazid, rifampicin, and ethambutol was initiated, taking into account that the patient had been receiving treatment for primary biliary cholangitis. The patient showed clinical improvement after 4 months of antituberculous therapy, and follow-up CT demonstrated a marked reduction in the size of the lymphadenopathies (Fig. 1c).

## Discussion

This case was diagnosed through a stepwise approach: initial confirmation of QFT positivity in the context of histopathological findings from abdominal lymph nodes obtained via EUS-TA, followed by definitive verification through *M. tuberculosis* culture.

In Japan, extrapulmonary tuberculosis constitutes a substantial proportion of tuberculosis cases, with tuberculous pleuritis being the most frequently encountered form. Among abdominal manifestations of tuberculosis, gastrointestinal tuberculosis and tuberculous peritonitis are observed more commonly, whereas abdominal tuberculous lymphadenitis is considered relatively uncommon. Clinically, abdominal tuberculous lymphadenitis typically presents with non-specific symptoms such as abdominal pain, weight loss, and fever.

Interferon-γ release assays (IGRAs), including QFT, measure interferon-γ responses to *M. tuberculosis*-specific antigens and are not affected by previous Bacillus Calmette-Guérin vaccination. Because of these characteristics, IGRAs are useful for evaluating prior tuberculosis exposure in patients with suspected tuberculous lymphadenitis.

In the present case, multiple low-density lymph node masses with solid rims were observed on contrast-enhanced CT, a radiologic finding that is considered characteristic of tuberculous lymphadenitis. This pattern corresponds to central caseous necrosis surrounded by peripheral active inflammation in enlarged lymph nodes infected with tuberculosis [3]. The IGRA results supported the suspicion raised by histopathological findings from the initial EUS-TA, prompting a second procedure aimed at obtaining samples for conducting microbiological tests.

Since histological confirmation of caseating granuloma or detection of *M. tuberculosis* in regional lymph nodes is the gold standard for diagnosing tuberculous lymphadenitis, histopathological examination of the affected lymph nodes is indispensable [3]. Because extrapulmonary tuberculosis is paucibacillary, accurate diagnosis of tuberculous lymphadenitis can be missed using Ziehl-Neelsen staining alone. The detection of *M. tuberculosis* by bacterial culture or PCR allows for a definitive diagnosis, even in the absence of histological evidence of tuberculosis.

Of the 33 reported cases of abdominal tuberculosis diagnosed by EUS-TA identified through searching PubMed for the period 2000–2025 using the keywords “tuberculous lymphadenitis EUS,” “tuberculous lymphadenitis EUS-FNA,” and “abdominal tuberculosis EUS,” including the present case, *M. tuberculosis* was confirmed in 14 (88%) by bacterial culture in 16 tests, in 15 (88%) by PCR in 17 tests, and in 16 (84%) by Ziehl-Neelsen staining in 19 tests, with some overlap among the cases. Of the cases diagnosed by bacterial culture, 5 (36%) were positive by bacterial culture alone as in the present case, 6 (75%) were positive by PCR in 8 tests, and 6 (67%) were positive by Ziehl-Neelsen staining in 9 tests [2–14] (Fig. 3). Although PCR reportedly has higher sensitivity than bacterial culture for tuberculous lymphadenitis (90.1% vs. 74.5%), there are cases in which tuberculosis was diagnosed solely by bacterial culture [15]. This suggests that including bacterial culture, in addition to PCR and Ziehl-Neelsen staining, may improve the diagnostic yield of tuberculosis.

**Fig. 3.**
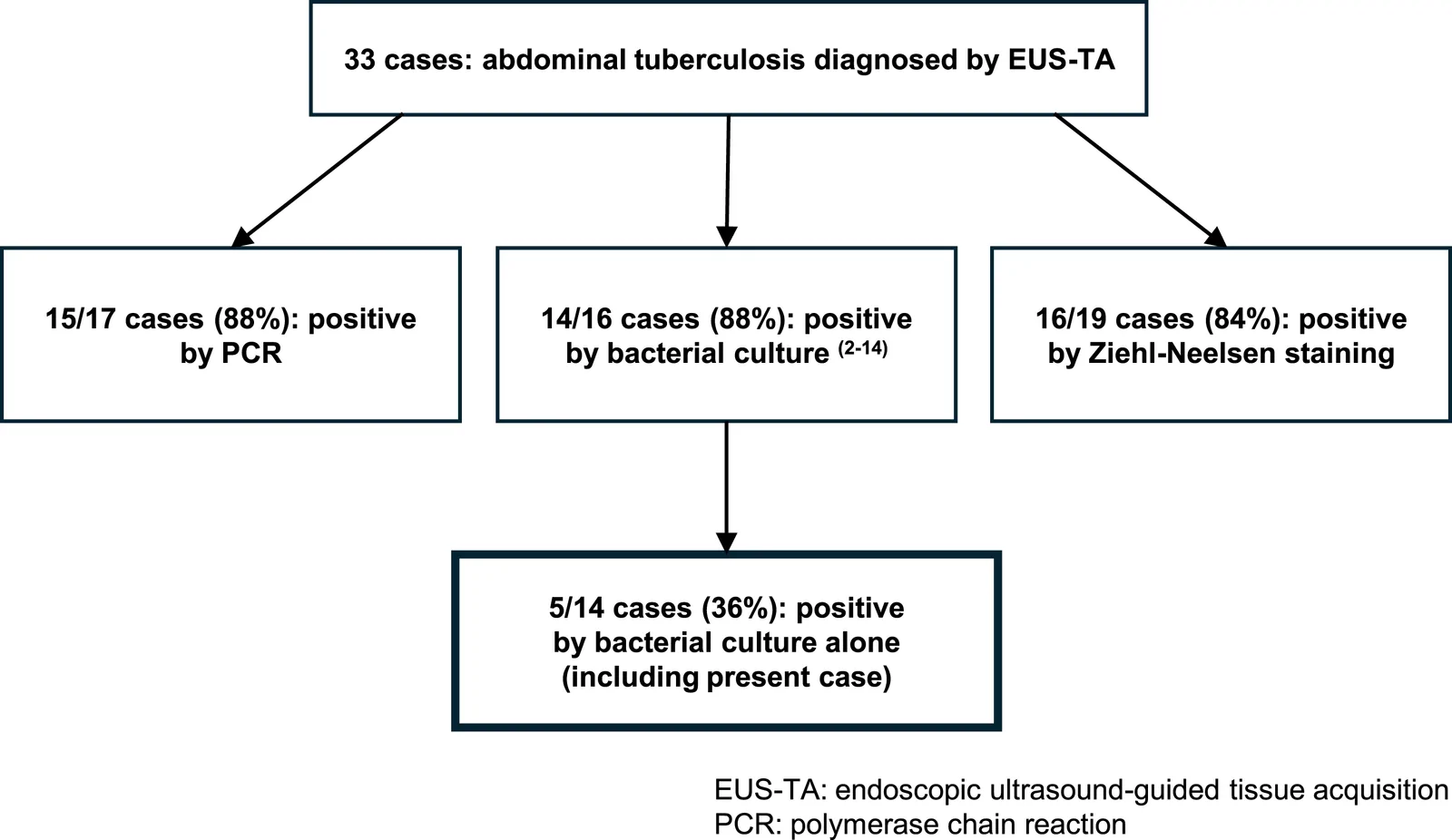
Of the 33 reported cases of abdominal tuberculosis diagnosed by EUS-TA identified through searching PubMed for the period 2000–2025 using the keywords “tuberculous lymphadenitis EUS,” “tuberculous lymphadenitis EUS-FNA,” and “abdominal tuberculosis EUS,” including the present case, *M. tuberculosis* was confirmed by bacterial culture in 14, polymerase chain reaction (PCR) in 15, and Ziehl-Neelsen staining in 16. Of the 14 cases diagnosed by bacterial culture, 5 were positive by bacterial culture alone, 6 were positive by PCR, and 6 were positive by Ziehl-Neelsen staining.

EUS-TA is a minimally invasive technique for obtaining histological samples from swollen abdominal lymph nodes [6]. The overall diagnostic accuracy of EUS-TA is approximately 89–92%. However, one study found that tuberculous lymphadenitis was diagnosed in only 83.1% of the cases [1]. This reduced yield is likely because aspirated material may contain necrotic debris with or without granulomas or giant cells and because extrapulmonary tuberculosis is typically paucibacillary. Performing not only histopathological examination but also bacterial culture and PCR is thought to enhance diagnostic accuracy and reduce the need for surgical intervention.

Differential diagnoses of abdominal granulomatous lymphadenopathy include sarcoidosis, nontuberculous mycobacterial infection, and fungal infection. Sarcoidosis is pathologically characterized by non-caseating granulomas and is frequently associated with pulmonary findings that may help distinguish it from tuberculosis. In the present case, the histopathological findings of necrotizing granulomas together with a positive IGRA result raised suspicion of tuberculous lymphadenitis, prompting repeat EUS-TA for microbiological testing. Given that a sufficient amount of sample was obtained during the initial EUS-TA, the same needle was used for the second procedure. A definitive diagnosis was established based on a positive bacterial culture from the specimen.

## Conclusion

We reported a case of abdominal tuberculous lymphadenitis diagnosed by repeated EUS-TA and bacterial culture, despite initially inconclusive histopathological findings. This case highlights the diagnostic limitations of histopathology alone and underscores the importance of combining bacterial culture and PCR with EUS-TA for the accurate diagnosis of granulomatous lymphadenopathy.

## Statement of Ethics

Ethical approval is not required for this case report in accordance with local or national guidelines. Written informed consent was obtained from the patient for publication of the details of their medical case and any accompanying images.

## Conflict of Interest Statement

The authors have no conflicts of interest to declare.

## Funding Sources

This study was not supported by any sponsor or funder.

## Author Contributions

Maiko Morii wrote this manuscript, contributed to the conception, and drafted of the article. Akie Yoshikawa-Kimura, Akinobu Nakata, Yoshinori Shimamoto, and Hirotsugu Maruyama carried out the diagnosis and treatment. Masahiro Kato and Masanori Shiohara were responsible for the histopathological evaluation and description. Yu Nishida, Masaki Ominami, Yuji Nadatani, Shusei Fukunaga, Koji Otani, Shuhei Hosomi, Fumio Tanaka, and Yasuhiro Fujiwara revised it critically for important intellectual content. Akie Yoshikawa-Kimura and Akinobu Nakata supervised this case report. All authors reviewed and approved the final manuscript.

## Data Availability

All data generated or analyzed during this study are included in this article and its online supplementary material files. Further inquiries can be directed to the corresponding author.
